# Supplementary material for: Integrated miRNA–mRNA Analysis Reveals Critical miRNAs and Targets in Diet-Induced Obesity-Related Glomerulopathy
Source: Int J Mol Sci. 2024 Jun 11;25(12):6437. doi: 10.3390/ijms25126437 (PMC11204096; doi:10.3390/ijms25126437)
Supplement: Supplementary file 1 [file ijms-25-06437-s001.zip › ijms-3016253_Supplementary Table S1.pdf]

International Journal of *Molecular Sciences*

MDPI

Supplementary Table S1. List ok key genes in heatmap 4b. This table provides the names and details of the key genes depicted in the heatmap in Figure 4b.

Ensembl ID	symbol	entrez	baseMean	log2FoldChange	lfcSE	pvalue	padj
ENSRNOG00000046975	Sult4a1	58953	241,098774	-1,340568632	0,347112507	4,0773E-06	0,01946838
ENSRNOG00000032740	Tmem258b	686092	252,642069	-0,531189784	0,203420206	0,00026477	0,44713276
ENSRNOG00000030016	Robo3	315564	15,0788239	-1,311382419	0,340522505	4,4211E-06	0,01946838
ENSRNOG00000002549	Htr5b	79247	29,4731203	-0,919285117	0,304082844	8,1404E-05	0,22188075
ENSRNOG00000015992	Ccl20	29538	23,7247145	1,717819412	0,543717709	5,258E-05	0,17198007
ENSRNOG00000003698	Mroh9	501864	19,5333366	1,14183749	0,687113415	0,00187957	0,80890823
ENSRNOG00000005964	Nr4a3	58853	21,0127717	1,252736725	1,301222559	0,00349861	0,95005312
ENSRNOG00000050420	Krt5	369017	82,9621309	2,906040377	2,223398859	0,00176233	0,80890823
ENSRNOG00000009350	Sez6	192247	8,4885964	1,108147742	0,998587835	0,00350442	0,95005312
ENSRNOG00000008513	LOC103690019	103690019	8,20616496	1,13364633	0,643098573	0,00169217	0,80890823
ENSRNOG00000016872	Plppr4	295401	18,7224694	1,014698008	0,388787374	0,00027341	0,44713276
ENSRNOG00000048723	Pros1	81750	650,261596	0,484378024	0,170879658	0,00014865	0,34728805
ENSRNOG00000030285	Epha3	29210	10,233124	1,831137002	0,472764972	4,7617E-06	0,01946838
ENSRNOG00000020685	Atp12a	171028	108,311016	1,69576889	0,431187891	3,1382E-06	0,01946838
