# Supplementary material for: Integrated miRNA–mRNA Analysis Reveals Critical miRNAs and Targets in Diet-Induced Obesity-Related Glomerulopathy
Source: Int J Mol Sci. 2024 Jun 11;25(12):6437. doi: 10.3390/ijms25126437 (PMC11204096; doi:10.3390/ijms25126437)
Supplement: Supplementary file 1 [file ijms-25-06437-s001.zip › ijms-3016253_Supplementary Table S2.pdf]

Supplementary Table S2. List of key differentially expressed smallRNA in urine heatmap 5c. This table provides the names and details of the key genes depicted in the heatmap in Figure 5c.

miRBase Alias	Name	Source	Biotype	baseMean	log2FoldChange	lfcSE	pvalue	padj
MIMAT0030409	rno-miR-155-5p	miRBase	miRNA	315,668744	1,0026939	1,25610312	0,00837661	0,12366929
ENSRNOT00000	U4.10-201	Ensembl	snRNA	61,5734488	1,115117904	0,97200326	0,00630156	0,10622775
ENSRNOT00000	SNORA62.6-201	Ensembl	snoRNA	37,2532645	1,084320988	2,28608821	0,00603297	0,10622775
ENSRNOT00000	U6.509-201	Ensembl	snRNA	20,1120311	1,324085182	0,80746964	0,00221455	0,06916198
ENSRNOT00000	U6.35-201	Ensembl	snRNA	18,6414419	1,429641308	0,83515942	0,00197702	0,06803481
ENSRNOT00000	U6.122-201	Ensembl	snRNA	35,5664982	1,11430092	0,92948618	0,00700518	0,11376405
ENSRNOT00000	U6.403-201	Ensembl	snRNA	47,2190805	0,981378365	0,98820164	0,00746525	0,11885843
MIMAT0000878	rno-miR-205	miRBase	miRNA	155,342795	1,826687269	0,88598175	0,00111361	0,05713903
MI0012597	rno-mir-615	miRBase	pre-miRNA	95,937334	-1,380185973	0,58744647	0,00119626	0,05713903
MIMAT0000832	rno-miR-126a-3p	miRBase	miRNA	30079,33	-1,03034411	0,44356132	0,00154875	0,06067761
MIMAT0000573	rno-miR-140-5p	miRBase	miRNA	457,828085	-1,164913242	0,36302287	0,00014766	0,04864462
ENSRNOT00000	AABR07070505.3-201	Ensembl	snoRNA	101,477847	-1,047402014	1,36047983	0,00968902	0,1249423
ENSRNOT00000	AABR07070505.4-201	Ensembl	snoRNA	101,477847	-1,047402014	1,36047983	0,00968902	0,1249423
MIMAT0017088	rno-let-7c-2-3p	miRBase	miRNA	523,359294	-1,330470495	0,58466228	0,00087364	0,05456914
MIMAT0017085	rno-let-7a-1-3p	miRBase	miRNA	523,26061	-1,331454077	0,58469181	0,00087077	0,05456914
MIMAT0000850	rno-miR-144-3p	miRBase	miRNA	161,038617	-1,2281105	1,05761174	0,00323218	0,07953126
MIMAT0000834	rno-miR-128-3p	miRBase	miRNA	250,792258	-1,164409032	0,44845165	0,00062659	0,04864462
MIMAT0000585_	rno-miR-340-3p	miRBase	miRNA	25,3193621	-1,317471512	0,46048438	0,0003581	0,04864462
ENSRNOT00000	SNORA74.5-201	Ensembl	snoRNA	39,5568102	-1,3133499	0,73090101	0,0026353	0,07242668
MIMAT0017090	rno-let-7f-2-3p	miRBase	miRNA	100,163311	-1,023007462	0,40960958	0,00117971	0,05713903
MIMAT0003152	rno-miR-22-5p	miRBase	miRNA	283,753621	-1,181488062	0,52799833	0,00133979	0,06043921
MIMAT0000800	rno-miR-28-5p	miRBase	miRNA	259,83096	-1,394472179	0,41554988	6,853E-05	0,04864462
ENSRNOT00000	SNORD16.2-201	Ensembl	snoRNA	4922,75601	-1,789942307	0,68175173	0,00046205	0,04864462
MI0021853	rno-mir-6329	miRBase	pre-miRNA	28,2515181	-0,563365718	0,32907522	0,01228715	0,13857178
MIMAT0037264	rno-miR-676	miRBase	miRNA	172,917183	-1,175247114	0,81457648	0,00448715	0,09823614
MIMAT0005337	rno-miR-760-3p	miRBase	miRNA	18,516439	-1,221691979	0,62332813	0,00288347	0,07316794
MIMAT0000902	rno-miR-300-3p	miRBase	miRNA	66,0170894	-1,061300051	0,52158523	0,00270548	0,07242668
MIMAT0000862	rno-miR-185-5p	miRBase	miRNA	766,866334	-1,006052468	0,35090019	0,00046246	0,04864462
