# Supplementary material for: Integrated miRNA–mRNA Analysis Reveals Critical miRNAs and Targets in Diet-Induced Obesity-Related Glomerulopathy
Source: Int J Mol Sci. 2024 Jun 11;25(12):6437. doi: 10.3390/ijms25126437 (PMC11204096; doi:10.3390/ijms25126437)
Supplement: Supplementary file 1 [file ijms-25-06437-s001.zip › ijms-3016253_Supplementary Table S3.pdf]

International Journal of
Molecular Sciences

MDPI

Supplementary Table S3. List of genes expressed in the rat kidneys of our animal model, illustrating interactions with miRNAs that were identified as differentially expressed (DE) in the rat kidneys.

Described regulated gene	Number of miR-mRNA interactions	miR ID
Abhd12b, Actl11, Armc6, Atcay, Atp13a4, B3gnt5, Ccnf, Celf4, Chgb, Cyp2a1, Cyp2a2, Cyp2a3, Dbndd1, Dmrt3, Dmrta2, Ereg, Fam71a, Frem3, Gbx2, Glp2r, Grm1, Grm6, Has2, Hcar2, Ido1, Iqcf3, Kctd4, Klfl4, Krt77, Ltb, egf10, Mpm13, Myh6, Notum, Nppa, Nppb, Oas1e, Odf1, Orl156, Orl210, Olr419, Orl455, Orl459, Orl72, Orl758, P4ha3, Pdzd9, Pou4f3, Romo1, Rps27a, Rspo2, S100a5, Scn9a, Serpinb7, Sh3bgrl2, Scl17a7, Scl25a54, Snap25, Snorc, Spem1, Tagln3, Tbx15, Tmem30c, Tnfsf4, Tnn, Vom2r75, Wfdc21, Xirp2, Yif1b	No miR interactions	
Acan, Abraxas1, Acap1, Acot7, Acp1, Acrbp, Acsm3, Actr3b, Actr5, Adm, Adora2b, Adpgk, Adprhl2, Adpm, Ahvy, Ahrr, Ak2, Akna, Alox15, Amdhd2, Amotl2, Anapc2, Ang2, Ankrd23, Ankrd34a, Ankrd50, Ankrd6, Ankrd9, Ap2s1, Apex1, Apln, Aqp1, Arhgap25, Arhgef7, Arid3b, Arsg, Atg7, Atrip, Avpi1, B3gnt5, Bach2, Banp, Bbs10, Bbs4, Bbs5, Bcar3, Bckdhb, Bcl2l2, Bcl3, Bco2, Bcor, Bcorl1, Begain, Best1, Bfsp1, Bmi1, Bmp2, Borcs6, Bphl, Brsk1, C4bpb, C8g, Cabp1, Cacna1d, Cacna2d2, Camkmt, Camlg, Cand2, Capg, Card10, Cbx2, Ccdc126, Ccdc62, Ccl4, Ccl5, Ccn2, Cct5, Cd24, Cd3eap, Cd46, Cd74, Cdk20, Cdk5rap1, Celf2, Ces2g, Cfap410, Cfap52, Cflar, Ch25h, Chrnb1, Chst15, Cisd3, Ckmt1, Clcf1, Cldn11, Cldn23, Cldn8, Cog1, Col11a2, Col18a1, Comtd1, Coprs, Coro1a, Cpne5, Cpne7, Cryzl1, Csf1, Csnk2b, Cuedc1, Cwf19l1, Cxcl1, Cxcl11, Cyp19a1, Cyp24a1, Cyp46a1, Cyth2, Dbp, Dcaf15, Dctn3, Dcun1d3, Ddx25 ,Deaf1, Dennd6b, Depdc1b, Dhodh, Dipk1b, Dll4, Dmac2, Dnaaf5, Dnajb13, Dohh, Dolpp1, Dpp10, Dpy30, Dtwd1, Dtx2, Dusp1, Dusp27, Dusp4, Dusp6, E2f3, Eci1, Efna4, Efnb1, Egfr, Egr2, Ehd4, Eif2d, Elf2, Elmsan1, Elp6, Eme1, Epha2, Ephb1, Ephb3, Fabp3, Fam110c, Fam193b, Fam214b, Fam221a, Fam89a, Fam89b, Fank1, Fbxl6, Fcgr3a, Fem1a, Fermt3, Fgf1, Fgr, Fhl3, Fjx1, Fmc1, Fmo4, Fmod, Fntb, Foxred2, Frmpd4, Frs3, Fscn3, Fsip1, Fyb1, G4, Gadd45b, Galnt2, Gapt, Gatc, Gen, Gfpt2, Ggt7, Gk, Gltp, Gmppb, Gpm6b, Gpr108, Gpr19, Gpr68, Gpt, Gpx8, Grb10, Grem2, Grid2, Grn, Gsdma, Gstk1, Gtf3c5, Gucy1b1, Hapln4, Harbi1, Hdac4, Hdhd3, Hint2, Hlx, Hmgn5b, Hoxc5, Hoxd9, Hps4, Hpx, Hras, Hsd17b13, Hsd17b8, Htr5b, Hyal2, Ifi30, Ift74, Igfbp5, Ighmbp2, Igsf6, Igsf9, Il1r2, Il22ra1, Ino80b, Insyn1, Iqub, Irak2, Irf2bpl, Irf3, Irs1, Isyna1, Itga8, Itgb1bp2, Itgb7, Itpka, Jmjd7, Jph2, Jun, Junb, Kank4, Kat8, Kcnd3, Kcnh6, Kcnn1, Kdm8, Kiaa0408L, Kif2c, Kin, Klc1, Klf16, Klf5, Klhl26, Kxd1, Lactb, Lamb3, Lcmt2, Lgals3, Limk2, Lipt2, Lmntd2, Lmo1, Lnx2, Lpxn, Lrfn4, Lrif1, Lrp2bp, Lrrc24, Lrrc59, Lrrc71, Lrrn4cl, Ltbr, Lxn, Lypla2, Lzts2, Maff, Mageh1, Map3k14, Map3k7cl, Map3k8, Mapk14, Mapkapk3, Masp1, Masp2, Mbip, Mblac1, Megf10, Mettl18, Mettl3, Mfsd11, Mfsd12, Micu3, Midn, Mier2, Mks1, Mlst8, Mob3a, Mon1b, Mrpl12, Mrpl14, Mrpl19, Mrpl21, Mrpl37, Mrpl49, Mrpl52, Mrps31, Mtfp1, Myd88, Myo7b, Myocd, Nans, Ncam2, Ncf1, Nckap1l, Ncln, Ndufaf8, Ndufv3, Nek8, Neurl4, Nfkbil1, Ngrn, Nme3, Nme7, Nmnat1, Nmrk1, Noc2l, Nod2, Nol12, Nol3, Notch1, Npr1, Nr1d1, Nr2c1, Nr2f2, Nr2f6, Nsmf, Nt5c1a, Ntn4, Nupl2, Ocel1, Odf2l, Opn3, Osgin1, P2rx7, P2ry4, P2ry6, Pafah1b3, Pafah2, Pag1, Paqr6, Parm1, Pcbd1, Pcdh18, Pcdhb8, Pclaf, Pcyox1l, Pde1c, Pde4b, Pde5a, Pdgfra, Pdia5, Pdxk, Pdzd7, Pex3, Pgm1, Phb2, Phc2, Phf1, Phrf1, Phykpl, Pih1d2, Pik3cd, Pik3ip1, Pim1, Pim3, Pitpnm1, Pkdcc, Plac9, Plcd3, Plec, Plet1, Plk2, Plp1, Plpp7, Pmel, Pnkd, Pnma1, Pnn, Podxl2, Polr1e, Polr2d, Ppara, Ppp1r32, Ppp1r36, Pqbp1, Prdm1, Prelid3a, Prr5 ,Prr5l, Prss23, Psd, Psma1, Ptcd1, Ptcd2, Ptdss2, Ptpn23, Ptpre, Ptx3, Pygm, Qrsl1, Rab12, Rab20, Rab33a, Rabep2, Rabggta, Rad17, Ralgds, Rap1gap2, Rbks, Rbm48k, Rcor1, Recql4, Rell2, Rft1, Rgl2, Rgma, Rgs2, Rhpn2, Riox1, Rmdn2, Rnf126, Rnf144a, Rnf225, Rnf24, Rnf32, Rnf39, Rnh1, Rnpc3k, Rpp21,Rpusd1, Rras, Rrnad1, Rsrp1, RT1-Da, Rundc3b, Ruvbl2, Rwdd2a, S100a3, S100b, Satb2, Sbno2, Scarb1, Scg2, Scx, Sema4a, Sema4b, Sema4d, Serinc4, Serpinf2, Sertad3, Sfxn2, Sgms1, Sirt1, Slamf8, Slc12a7, Slc22a17, Slc25a10, Slc25a22, Slc25a39, Slc26a6, Slc35e3, Slc35e4, Slc35f6, Slc37a1, Slc46a3, Slc4a11, Slco2a1, Smad7, Smim11a, Smyd1, Snrnp48, Snrpd1, Socs3, Sowahc, Sox13, Spata24, Spata5l1, Speg, Spin2a, Spint1, Spry1, Srsf5, Srsf9, Stk16, Sts, Sult1a1, Sult2b1, Synpr, Syt7, Sytl4, Szrd1, Tagln3, Tbc1d25, Tbp, Tbx3, Tefm, Thap4, Thbd, Thegl, Thnsl2, Timm10, Tle2, Tlr2, Tlr7, Tmem130, Tmem134, Tmem14c, Tmem186, Tmem19, Tmem202, Tmem26, Tmem53, Tnfaip2, Tnfrsf14, Tnfrsf1b, Tnfsf10, Tnip2, Tomm40l, Tonsl, Tor2a, Tp53i3, Tp53inp1, Tpgs1, Tpst2, Traf2, Trappc4, Trib2, Trim11, Trim3, Trim47, Trim5, Trmt5, Trpm4, Tsen15, Tsnaxip1, Tspan18, Tuba4a, Txnrd2, Tymp, U2af1l4, Uba7, Ubd, Ube2q2l, Ube3d, Ubox5, Ubtd1, Ugt8, Unc119, Urb2, Urm1, Usb1, Usp43, Usp44, Utp23, Vav2, Vcam1, Vcan, Vim, Vsir, Vwa3a, Vwa7, Wdr19, Wdr24, Wdsub1, Wnk1, Wnt5b, Xcr1, Xkr8, Xkr9, Zbtb22, Zfp217, Zfp354c, Zfp384, Zfp394, Zfp418, Zfp458, Zfp46, Zfp57, Zfp653, Zfp786, Zfp787, Zmat4, Znhit2	1 miR interactions	miR-140-5p miR-185-5p miR-28-5p miR-340-3p miR-6329 miR-674-3p miR-148b-5p
Acadsb, Arl5c, Bid, Ccnf, Cd83, Cdo1, Ceacam4, Cr11, Cx3cl1, Cxcl2, Edn1, Hgf, Kcnk5, LOC103689942, Lrp4, Myh6, Myh7, Nfkbia, Nppa, Plau, Pop7, RGD1309808, Rexo1, Rhbdf2, Sec61g, Sgk1, Slc25a22, Slc29a1, Snai1, Tnf	2 miR interactions	miR-125b-5p miR-128-3p
Pten	4 miR interactions	miR-22-3p miR-22-5p miR-144-3p miR-205
