# Supplementary material for: Integrated miRNA–mRNA Analysis Reveals Critical miRNAs and Targets in Diet-Induced Obesity-Related Glomerulopathy
Source: Int J Mol Sci. 2024 Jun 11;25(12):6437. doi: 10.3390/ijms25126437 (PMC11204096; doi:10.3390/ijms25126437)
Supplement: Supplementary file 1 [file ijms-25-06437-s001.zip › ijms-3016253_Supplementary Table S4.pdf]

International Journal of *Molecular Sciences*

MDPI

Supplementary Table S4. List of key differentially expressed smallRNA in heatmap 8. This table provides the names and details of the key smallRNA in urine depicted in the heatmap in Figure 8.

miRBase Alias	Name	Source	Biotype	baseMean	log2FoldChange	lfcSE	pvalue	padj
MIMAT0000781_1	rno-miR-9a-5p	miRBase	miRNA	4,36912814	2,760965633	0,85365666	8,76705E-05	0,004938769
MIMAT0000820	rno-miR-99a-5p	miRBase	miRNA	70,3367145	2,509256337	0,594591499	1,55414E-06	0,000787951
MIMAT0000888	rno-miR-218a-5p	miRBase	miRNA	6,76043916	2,481556687	0,736431258	7,03002E-05	0,004613157
MIMAT0000615	rno-miR-101b-3p	miRBase	miRNA	7,7583044	2,412736788	0,652629825	1,31981E-05	0,002230478
MIMAT0000805	rno-miR-30e-5p	miRBase	miRNA	71,0422035	2,375273586	0,692136473	3,81674E-05	0,003870179
MIMAT0000823	rno-miR-101a-3p	miRBase	miRNA	20,8497583	2,320777039	0,67334924	3,62833E-05	0,003870179
MIMAT0000779	rno-let-7i-5p	miRBase	miRNA	89,3850692	2,222078531	0,574294438	7,32052E-06	0,001855753
MIMAT0000808	rno-miR-30a-5p	miRBase	miRNA	335,147551	2,1671462	0,639592697	4,76727E-05	0,00402834
MIMAT0000799	rno-miR-27a-3p	miRBase	miRNA	9,28988705	2,157366396	0,727175003	0,000250177	0,007523755
MIMAT0000803	rno-miR-29c-3p	miRBase	miRNA	6,03847862	2,002778183	1,027331691	0,002387968	0,031043578
MIMAT0005595	rno-miR-146b-5p	miRBase	miRNA	3,34456891	1,974302664	0,975076763	0,003077045	0,033991492
MIMAT0000602	rno-miR-20a-5p	miRBase	miRNA	5,44263763	1,941668089	0,754295839	0,000682587	0,015046597
MIMAT0001082	rno-miR-196b-5p	miRBase	miRNA	4,95567688	1,940102953	0,801517572	0,001222676	0,022959134
MIMAT0000878	rno-miR-205	miRBase	miRNA	10,0699557	1,913730055	0,68603692	0,000494307	0,012323541
MIMAT0000783	rno-miR-10b-5p	miRBase	miRNA	120,712152	1,890979382	0,645071678	0,000251857	0,007523755
MIMAT0000876	rno-miR-203a-3p	miRBase	miRNA	37,9049456	1,778676843	0,64995987	0,000510443	0,012323541
MIMAT0000802	rno-miR-29a-3p	miRBase	miRNA	83,6381514	1,757114761	0,655949332	0,000583451	0,013445885
MIMAT0000778	rno-let-7f-5p	miRBase	miRNA	191,738949	1,713602531	0,709399608	0,001199448	0,022959134
MIMAT0005442	rno-miR-30c-2-3p	miRBase	miRNA	5,38152588	1,660691713	0,794553096	0,002554802	0,031579814
MIMAT0000830_1	rno-miR-125b-5p	miRBase	miRNA	4,63499486	1,632321913	0,769486803	0,002709208	0,031943456
MIMAT0024847	rno-miR-1843a-5p	miRBase	miRNA	3,45501454	1,621328484	0,679461997	0,001852694	0,028862378
MIMAT0000819	rno-miR-98-5p	miRBase	miRNA	5,1163622	1,601418185	0,69120844	0,001745157	0,028541769
tRNA-Phe-GAA-1-1	chr1.trna8484	GtRNAdb	tRNA	79,1706292	1,571235383	0,796789985	0,003635429	0,036944797
tRNA-Phe-GAA-1-4	chr15.trna3836	GtRNAdb	tRNA	106,126921	1,543359319	0,64765946	0,001559897	0,026362267
MIMAT0000818	rno-miR-96-5p	miRBase	miRNA	2,61142515	1,528610538	0,828675294	0,005952176	0,049471366
MIMAT0003208	rno-miR-374-5p	miRBase	miRNA	1,93532689	1,517103342	0,966859919	0,008324771	0,064933213
MIMAT0000798	rno-miR-27b-3p	miRBase	miRNA	56,1409131	1,423682981	0,596222502	0,001878616	0,028862378
MIMAT0000606_1	rno-miR-7a-5p	miRBase	miRNA	12,5624092	1,412685551	0,611795714	0,002127407	0,02940875
MIMAT0000774	rno-let-7a-5p	miRBase	miRNA	135,902109	1,369007919	0,714983802	0,005307171	0,047079428
MIMAT0003381	rno-miR-499-5p	miRBase	miRNA	11,9852234	1,364001549	0,73389101	0,006386245	0,052223003
MIMAT0000790	rno-miR-21-5p	miRBase	miRNA	370,818563	1,34605202	0,666439594	0,004701655	0,044976206
tRNA-Arg-TCT-3-1	chr2.trna7920	GtRNAdb	tRNA	73,7173061	1,34353587	0,621677777	0,003366345	0,035557024
MIMAT0003379	rno-miR-378a-3p	miRBase	miRNA	219,98137	1,334474376	0,65726235	0,004525753	0,044126087
MIMAT0000846	rno-miR-141-3p	miRBase	miRNA	3,88858253	1,289394206	0,878681507	0,011302167	0,079877388
MIMAT0000777	rno-let-7e-5p	miRBase	miRNA	9,22578656	1,288072659	0,904882937	0,011756318	0,081650046
MIMAT0000782	rno-miR-10a-5p	miRBase	miRNA	237,277367	1,267791346	0,641022545	0,005611526	0,047417393
MIMAT0035719	rno-let-7g-5p	miRBase	miRNA	144,464009	1,172613292	0,700854559	0,011167177	0,079877388
MIMAT0035725	rno-miR-148a-3p	miRBase	miRNA	155,867828	1,171774572	0,835084298	0,015884618	0,104590927
MIMAT0000797	rno-miR-26b-5p	miRBase	miRNA	46,7576494	1,141093082	0,641297256	0,010141105	0,07673941
tRNA-Phe-GAA-1-5	chr17.trna4423	GtRNAdb	tRNA	110,161369	1,126317018	0,638422357	0,010640412	0,07933366
MIMAT0003152	rno-miR-22-5p	miRBase	miRNA	3,55179316	1,086486681	0,746375518	0,017945857	0,114095829
MIMAT0000775	rno-let-7b-5p	miRBase	miRNA	149,8883	1,061585197	0,769730436	0,02034863	0,125321713
MIMAT0001538	rno-miR-429	miRBase	miRNA	22,7314431	1,033147247	0,769670472	0,022235459	0,131085788
tRNA-iMet-CAT-2-1	chr17.trna4518	GtRNAdb	tRNA	6,04839695	1,030629536	0,531596014	0,009738183	0,074806948
MIMAT0000874	rno-miR-200a-3p	miRBase	miRNA	171,087	1,024318806	0,704852876	0,020763361	0,125321713
tRNA-Val-CAC-3-1	chr17.trna1814	GtRNAdb	tRNA	165,102134	-1,000170188	0,591958894	0,015246365	0,101709304
tRNA-Leu-CAA-2-1	chr10.trna13768	GtRNAdb	tRNA	82,1469327	-1,003108527	0,450624757	0,005478671	0,047079428
ENSRNOT00000063645	5S_rRNA.89-201	Ensembl	rRNA	32,8573067	-1,030767694	0,670208968	0,018587762	0,116345622
tRNA-Ser-AGA-5-1	chr17.trna4477	GtRNAdb	tRNA	1,88196163	-1,043680687	0,844345484	0,021215811	0,126546074
ENSRNOT00000089489	U1.42-201	Ensembl	snRNA	15,0844163	-1,06729102	0,78242395	0,02059494	0,125321713
ENSRNOT00000053460	5S_rRNA.232-201	Ensembl	rRNA	1,28640813	-1,159373266	1,14230862	0,023404922	0,134844266
tRNA-Lys-TTT-3-1	chr17.trna4472	GtRNAdb	tRNA	63,5276317	-1,162015328	0,701289601	0,011212191	0,079877388
tRNA-Ser-AGA-2-1	chr17.trna1843	GtRNAdb	tRNA	20,5869605	-1,193853075	0,516037755	0,003003256	0,033991492
tRNA-Lys-CTT-1-6	chr10.trna15534	GtRNAdb	tRNA	17,5162427	-1,217580268	0,556296279	0,003888939	0,038660633
tRNA-Leu-CAG-3-2	chr13.trna2774	GtRNAdb	tRNA	9,72535507	-1,277111277	0,582701343	0,003643471	0,036944797
tRNA-Ser-TGA-1-1	chr17.trna1849	GtRNAdb	tRNA	5,07977049	-1,321560889	0,690893101	0,005374743	0,047079428
ENSRNOT00000073261	Rn5s-201	Ensembl	rRNA	15,7658993	-1,397348778	0,808562639	0,00697534	0,055257771
ENSRNOT00000081846	5S_rRNA.277-201	Ensembl	rRNA	223,244422	-1,398135388	0,753131673	0,005339205	0,047079428
ENSRNOT00000086749	5S_rRNA.269-201	Ensembl	rRNA	150,229814	-1,411823477	0,810000931	0,006639521	0,053432338
ENSRNOT00000052818	5S_rRNA.15-201	Ensembl	rRNA	2,79061405	-1,438747736	1,036159573	0,011343534	0,079877388
tRNA-Lys-CTT-1-7	chr10.trna15529	GtRNAdb	tRNA	74,6863996	-1,527105713	0,591480714	0,000899145	0,01899443
ENSRNOT00000088446	SNORD14.1-201	Ensembl	snoRNA	55,6576579	-1,560593869	0,761231728	0,003151085	0,033991492
ENSRNOT00000090754	SNORD14.4-201	Ensembl	snoRNA	57,9379877	-1,628180378	0,725380885	0,001938769	0,028910464
tRNA-Ala-AGC-3-1	chr17.trna4448	GtRNAdb	tRNA	26,5004237	-1,752557949	0,810731937	0,002204206	0,02940875
tRNA-Ala-AGC-3-2	chr17.trna4437	GtRNAdb	tRNA	26,5004237	-1,752557949	0,810731937	0,002204206	0,02940875
ENSRNOT00000062405	5S_rRNA.52-201	Ensembl	rRNA	5,74105462	-1,870555321	0,805492095	0,001338354	0,023464825
tRNA-Asp-GTC-3-1	chrX.trna7882	GtRNAdb	tRNA	91,0817245	-2,026653192	0,673908376	0,000180524	0,007039089
tRNA-Val-CAC-1-2	chr10.trna1517	GtRNAdb	tRNA	17,9274053	-2,210400424	0,785784856	0,000280638	0,007904647
ENSRNOT00000070518	5S_rRNA.207-201	Ensembl	rRNA	32,2345777	-2,237293049	0,784630361	0,000252276	0,007523755
ENSRNOT00000085375	U6.44-201	Ensembl	snRNA	11,2913608	-2,345988305	0,887382553	0,000395364	0,010549975
tRNA-Pro-CGG-1-3	chr13.trna4827	GtRNAdb	tRNA	12,2408872	-2,354587083	0,762263149	0,000130196	0,006600934
ENSRNOT00000084401	U6.709-201	Ensembl	snRNA	12,3602559	-2,462608104	0,857615399	0,000194373	0,007039089
